# GeoAssemble: A Geometry-Aware Hierarchical Method for Point Cloud-Based Multi-Fragment Assembly

**DOI:** 10.3390/s25216533

**Published:** 2025-10-23

**Authors:** Caiqin Jia, Yali Ren, Zhi Wang, Yuan Zhang

**Affiliations:** 1 School of Computer Science and Technology, North University of China, Taiyuan 030051, China; 2 Shanxi Province Key Laboratory of Machine Vision and Virtual Reality, Taiyuan 030051, China; 3 Shanxi Vision Information Processing and Intelligent Robot Engineering Research Center, Taiyuan 030051, China

**Keywords:** feature representation, fracture point identification, fragment assembly, geometry-centered transformation mechanism

## Abstract

Three-dimensional fragment assembly technology has significant application value in fields such as cultural relic restoration, medical image analysis, and industrial quality inspection. To address the common challenges of limited feature representation ability and insufficient assembling accuracy in existing methods, this paper proposes a geometry-aware hierarchical fragment assembly framework (GeoAssemble). The core contributions of our work are threefold: first, the framework utilizes DGCNN to extract local geometric features while integrating centroid relative positions to construct a multi-dimensional feature representation, thereby enhancing the identification quality of fracture points; secondly, it designs a two-stage matching strategy that combines global shape similarity coarse matching with local geometric affinity fine matching to effectively reduce matching ambiguity; finally, we propose an auxiliary transformation estimation mechanism based on the geometric center of fracture point clouds to robustly initialize pose parameters, thereby improving both alignment accuracy and convergence stability. Experiments conducted on both synthetic and real-world fragment datasets demonstrate that this method significantly outperforms baseline methods in matching accuracy and exhibits higher robustness in multi-fragment scenarios.

## 1. Introduction

Fragment assembly technology is an important research direction in the fields of computer vision and intelligent manufacturing, with broad application value in scenarios such as cultural relic restoration [1], medical image analysis [2], and industrial quality inspection [3]. Traditional methods rely on hand-crafted geometric features for fracture surface matching, yet they often fail in complex fracture patterns due to insufficient feature generalization. In recent years, deep learning has effectively improved feature robustness through data-driven strategy, bringing new insights to fragment assembly research. Current research primarily follows two paths: semantic fragment reconstruction [4,5,6], which constrains the solution space through predefined fragment functional labels [7], and geometric fracture reconstruction, which relies solely on local geometric features to achieve prior-free reconstruction [8,9]. Although datasets such as PartNet [7] and Breaking Bad [8] have driven progress in this field, existing methods still encounter significant challenges in terms of accuracy and efficiency [10,11].

Currently, fragment assembly technology has achieved significant progress. Early methods depending on hand-crafted descriptors [12] have gradually been replaced by deep learning methods [13,14,15]. These methods leverage neural networks to extract more generalizable global or local features from fragments, thereby providing robust support for accurate matching. Matching strategies have become increasingly mature, with staged methods gradually becoming the mainstream approach: Initially, global shape features are utilized for efficient coarse matching to reduce the search space [16,17]. Subsequently, local descriptors integrated with attention mechanisms are applied to refine the matching process, thus effectively capturing the complex inter-fragment relationships. Furthermore, significant advancements have been achieved in the global alignment. Traditional methods relying on random initial transformations have been superseded by approaches that integrate geometric constraints and robust optimization algorithms. These advancements have substantially improved both the accuracy and stability of multi-fragment assembly [18,19].

Although existing methods have made significant progress in relevant fields, several critical limitations still persist and require urgent attention. First, feature extractors struggle to comprehensively represent the complex geometric structures of fragments. For example, PointNet++ constructs local neighborhoods using fixed strategies. However these strategies cannot adaptively capture the multi-scale features inherent in the geometric structure of irregular fracture surfaces, thereby leading to increased matching ambiguity. Second, single-stage matching encounters combinatorial complexity issues owing to the absence of global geometric constraints, while purely local matching is prone to interference from similar fracture patterns [17]. Third, random initialization may give rise to cumulative errors in symmetric structures or noisy scenes. The root cause of these issues lies in the ineffective integration of local geometric features and global distributions, as well as the lack of physically plausible constraints during the optimization process.

**Figure 1:**
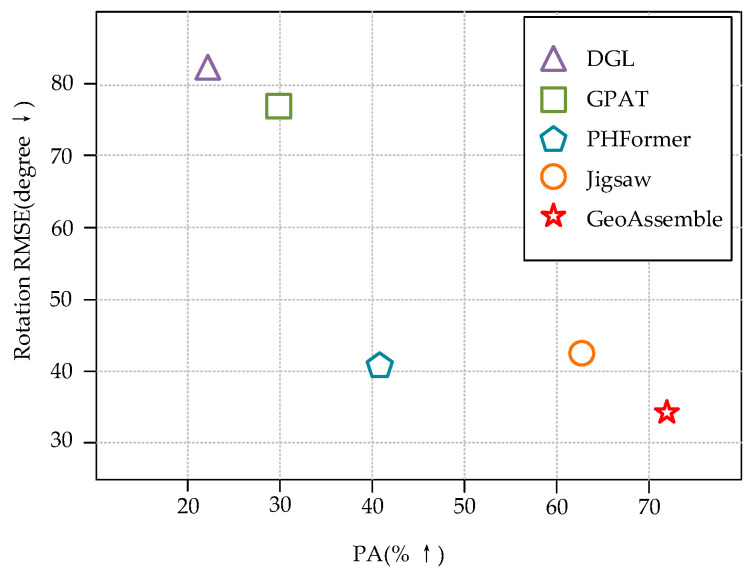
Experimental results from the Breaking Bad dataset compared the root mean square error (RMSE) and part accuracy (PA) between our proposed method and baseline approaches. The X-axis represents PA values (↑ indicates that higher values are better), while the Y-axis shows RMSE values (↓ indicates that lower values are better). Our method demonstrated significant reduction in RMSE while maintaining high assembly precision.

To address the above challenges, this paper proposes the GeoAssemble framework, a geometry-aware hierarchical assembly method that achieves high-precision multi-fragment assembly by synergistically integrating feature extraction and optimization strategies. The core idea of this work is that robust fragment assembly can be achieved via a geometry-driven pipeline independent of semantic information, thereby offering a more generalizable solution across diverse fracture types and object categories. Critically, the framework seamlessly balances data-driven generalization with strong geometric priors by embedding inherent physical properties (e.g., centroid-relative position, concavity/convexity) directly into its learnable feature encoding and matching pipeline. As shown in Figure 1, GeoAssemble significantly outperforms existing methods on the Breaking Bad dataset. It can effectively restore the complete geometric structure of complex fracture objects and exhibits stronger robustness in noisy and multi-fragment scenarios. The core contributions of this paper include:

- Geometry-Enhanced Feature Encoder: We develop a DGCNN-based feature extractor that integrates centroid-relative position features to construct a multi-dimensional feature representation. This innovation effectively addresses the challenge of multi-scale adaptability on irregular fracture surfaces.
- Global-Local Hierarchical Matching Mechanism: We design a two-stage matching strategy that integrates global geometry-guided coarse matching with local geometry-constrained fine matching. This strategy incorporates differentiable optimization and dynamic weight learning mechanisms, significantly reducing combinatorial ambiguity.
- Geometry-Aware Auxiliary Transformation Generation: We propose a physically plausible approach for generating an auxiliary transformation, specifically aimed at revising initial pose transformation. This approach effectively prevents error accumulation from random initialization.

Unlike previous methods requiring semantic labels or limited to pairwise matching, GeoAssemble operates category-agnostically and supports scalable multi-fragment assembly. This synergistic combination provides a robust, annotation-free solution that significantly pushes forward the state-of-the-art in fracture assembly.

## 2. Related Work

Research on point cloud-based fragment assembly mainly focuses on four key aspects: feature matching, semantics-based assembly, geometry-based assembly, and low-overlap point cloud registration. Recent advancements in deep learning have driven significant progress across these domains.

### 2.1. Feature Matching

Feature matching seeks to establish correspondences between fracture surfaces based on geometric consistency. Early methods relied on hand-crafted features (e.g., curvature and normal vectors) for local matching, yet suffered from noise sensitivity and limited generalization for complex fractures [1]. Recent deep learning methods utilized convolutional neural networks (CNNs) and graph neural networks (GNNs) to extract multi-scale geometric features, demonstrating superior performance in image registration [20,21] and multi-view matching [22,23,24]. However, existing methods are mostly tailored for pairwise fragment matching, and encounter the combinatorial explosion problem (exponential growth of the search space) when dealing with multi-fragment scenarios. Moreover, they lack explicit modeling of global geometric constraints. Additionally, methods based on global features [15] ignore local details, leading to heightened matching ambiguity as the number of fragments increases.

### 2.2. Semantics-Based Assembly

Semantics-based methods guide assembly utilizing predefined feature labels, such as connection surfaces, support structures, and achieve satisfactory performance on structured objects (e.g., furniture, mechanical parts). For example, PartNet [7] generates fragment labels via semantic segmentation and combines graph neural networks to predict assembly order. However, such methods heavily rely on high-quality semantic annotation data and cannot handle scenarios with random fractures lacking clear semantic information (e.g., archaeological fragments or naturally broken objects) [25]. Additionally, the high cost of annotating semantic labels limits their industrial applications [26]. Some studies have attempted to reduce annotation dependency through weakly supervised learning [27], but their performance still lags significantly behind fully supervised methods [28].

### 2.3. Geometry-Based Learning Methods

Geometry-based learning methods significantly improve assembly robustness by integrating geometric features with deep learning. Traditional methods rely on manual features (such as integral invariants and normal vector clustering) to segment fracture surfaces, they exhibit limited performance under noisy conditions and complex geometric interference. In contrast, deep learning methods automatically extract multi-scale features utilizing point cloud networks (e.g., PointNet [29], DGCNN) and optimize the assembling process through symmetry detection. For example, RoReg [30] proposed a geometric Transformer that enforces local rotation invariance, boosting registration accuracy in low-overlap regions. The SE(3) equivariant network [31] achieves fragment pose regression through symmetry constraints, significantly improving initialization robustness. Shape prior learning methods, including Jigsaw++ [32] and FragmentDiff [33], encoded the geometric distribution of the whole-object via implicit generative models to guide fragment alignment. However, balancing geometric priors with data-driven generalization remains a research challenge.

### 2.4. Low-Overlap Point Cloud Registration

Low-overlap point cloud registration remains a core challenge in fragment assembly. Traditional methods like ICP [34] and RANSAC rely on initial alignment assumptions, yet the small overlap between fragments often leads to convergence in local optima. Deep learning methods break these limitations by jointly optimizing feature matching and pose estimation: Geometric Transformer [35] designs a geometry-aware attention mechanism to enhance the local features discrimination; DiffAssemble [36] combines a diffusion model for pose denoising with Transformer iterative optimization for multi-fragment poses. Additionally, graph optimization methods based on cyclic consistency enforce global consistency through low-rank matrix recovery constraints. However, existing methods lack adaptability to degraded fracture surfaces (e.g., erosion, missing parts) and require integration of physical priors and multimodal information to enhance generalization.

### 2.5. Summary

In summary, current learning-based fragment assembly methods still face several critical gaps: (1) Limited Feature Adaptivity: Fixed-scale feature extractors struggle to represent multi-scale geometric structures of irregular fractures; (2) Matching Ambiguity: Single-stage matching or purely local/global strategies suffer from combinatorial explosion or ambiguity, especially in multi-fragment scenarios; (3) Initialization Sensitivity: Pose estimation often depends on random initialization, leading to error accumulation in symmetric or noisy structures; (4) Semantic Dependency: Many high-performing methods require semantic annotations, limiting their applicability in prior-free scenarios like archaeological restoration.

The GeoAssemble framework is explicitly designed to fill these gaps. It introduces a geometry-aware hierarchical approach that: leverages dynamic graph CNNs and centroid-relative encoding to enhance feature representation (addressing Gap 1); employs a two-stage matching strategy to reduce search space and refine correspondences (addressing Gap 2); utilizes a fracture-region geometric center for robust initialization (addressing Gap 3); and operates without semantic labels, relying solely on geometric cues for generalizable assembly (addressing Gap 4).

## 3. Methods

Given a set of fragmented point cloud data P = {P_1_, P_2_, …, Pₙ}, the objective is to recover the complete object O = T_1_ (P_1_) ∪ T_2_ (P_2_) ∪ … ∪ Tₙ (Pₙ), where *T_i_* denotes the spatial transformation applied to the i-th fragment *P_i_*. Under the assumption of rigid fracture, the original object O does not undergo deformation, and each fragment is precisely aligned through rigid body transformations.

**Figure 2:**
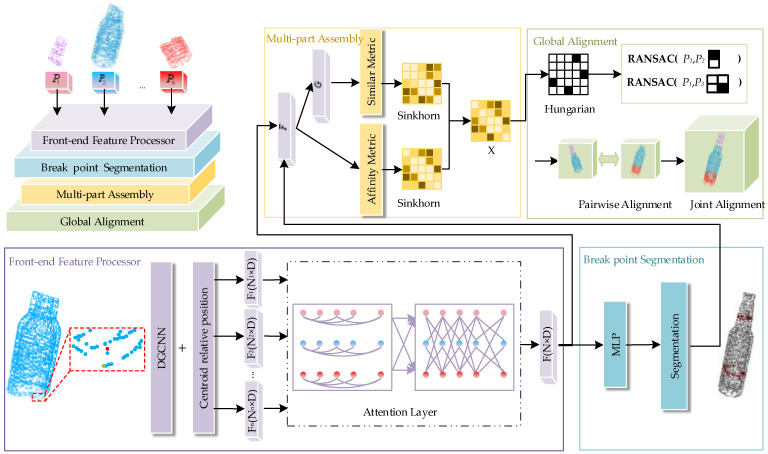
Overall network structure of GeoAssemble. This framework consists of four parts: The front-end feature extractor utilizes a DGCNN that fuses centroid relative position features (enlarged points in the figure: green for center point, blue for neighboring points, red for centroid point; centroid relative position reflects surface concavity/convexity), and combines self-attention and cross-attention to extract features for each point. The segmentation module identifies fracture points, while multi-fragment assembly establishes the correspondence between fracture points across multiple fragments. Global alignment is divided into pairwise transformation alignment and global pose estimation.

The GeoAssemble framework implements end-to-end fragment assembly through four modules, as shown in Figure 2. The front-end feature extraction module integrates the centroid relative position features to construct a dynamic neighborhood graph using DGCNN. By combing self-attention and cross-attention mechanisms, the module effectively captures long-range dependencies and improves the fragments geometric representation. The break point segmentation module separates the fracture surface from the original surface using a binary classifier based on learned features. The multi-fragment Assembly module adopts a two-stage strategy combining global coarse matching and local fine matching. The global alignment module is divided into two parts: pairwise transformation alignment and global pose estimation. Details of each part will be discussed below.

The entire framework is optimized in an end-to-end manner using a multi-task joint loss: the segmentation loss adopts a weighted cross-entropy function, the matching loss utilizes bidirectional contrastive constraints, and the pose loss ensures geometric consistency through SE(3) equivariant loss functions. Experiments on the synthetic and real datasets demonstrate that this framework significantly improves assembling accuracy. By synergistically optimizing feature extraction and hierarchical matching, this method provides a high-precision, high-efficiency solution for practical applications such as digital restoration of cultural heritage.

### 3.1. Front-End Feature Extractor

In this study, the front-end feature extractor designs a deeply integrated architecture based on dynamic graph convolutional networks and geometric attention mechanisms, aiming to significantly improve the accuracy of fracture point identification. This architecture achieves a refined point cloud representation by leveraging local feature aggregation and cross-fragment relationship modeling. The input point cloud $P\in R^{N\times 3}$ (where N is the number of points) undergoes multi-level feature extraction via dynamic graph convolutional module. Specifically, a dynamic neighborhood is constructed for each point using the kNN algorithm. For each neighborhood, the following 3D geometric information is computed: the coordinate of the central point *p*, the relative coordinates of neighboring points $p_{j}-p$, and the relative position of the neighborhood centroid $\frac{1}{k}\sum_{j}(p_{j})-p$, which is a crucial descriptor for capturing the local concavity or convexity of the fracture surface (as visually emphasized in Figure 2). These features are subsequently concatenated to form a locally augmented representation, which can be expressed as

(1) $$F(p)=Concat(p_{j}-p,p,\frac{1}{k}\sum_{j}(p_{j})-p),$$

Here, $p_{j}$ denotes the coordinate of the j-th neighbor point; *k* is the number of neighborhood points. Subsequently, four 1D convolution layers and max pooling operations are applied to extract features with 64, 64, 128, and 256 channels, respectively. Finally, these features are integrated via skip connections into a 512-dimensional point-wise feature $f_{p}\in R^{512}$.

To better capture fine-grained geometric features and enhance representation ability, this paper employs a point Transformer layer for spatially aware attention modeling of input features. This layer maps the point-wise features into query vector $q_{p}=W_{Q}f_{p}$, key vectors $k_{p}=W_{K}f_{p}$, and value vectors $v_{p}=W_{V}f_{p}$. Subsequently, it integrates these vectors with the MLP that encodes relative positions $\phi_{pos}(p_{j}-p)$ to generate geometry-sensitive attention weights, formulated specifically as:

(2) $$\alpha_{ij}=softmax((q_{i}^{T}k_{j}+\emptyset_{pos}(p_{j}-p))/\sqrt{d}),$$

Here, *W_Q_*, *W_K_*, *W_V_* are the projection weight matrices for the query, key, and value vectors, respectively; *ϕ_pos_* is the position encoding MLP; and *d* is the feature dimension scaling factor.

By weighting and aggregating neighborhood features and integrating residual connections, the network adeptly embeds the concavity details and geometric continuity of the fracture surface greatly enhancing the representation ability of local geometric structures. Furthermore, the cross-fragment attention layer establishes global associations among fragments via a multi-head mechanism and utilizes a position feedforward network to expand the nonlinear expression capability of features, jointly providing crucial feature support for accurate fracture point identification.

### 3.2. Break Point Segmentation

Compared to 3D registration methods, this paper emphasizes identifying the contact regions between fragments, which is critical for accurate assembly. The geometric properties of these contact regions guarantee perfect fitting when correctly matched. Therefore, the performance of the segmentation module directly determines the upper bound of overall assembly accuracy. However, real-world point clouds are discontinuous and noisy, making it difficult for manual feature-based methods to accurately segment the fracture surface. Therefore, this method employs a deep learning model to directly learn discriminative features from the data, thereby converting the fracture surface identification problem into a binary classification task for efficient and accurate segmentation.

Given an input point cloud $P\in R^{N\times 3}$, the segmentation module aims to predict the probability $c_{p}\in [0,1]$ that each point p∈P belongs to the fracture surface. Let $P_{i}$ be the point cloud of the i-th fragment, and $P_{i}^{f}\in P_{i}$ denote the set of points lied on its fracture surface. For any two adjacent fragments $P_{i}$ and $P_{j}$, the intersection of their fracture surfaces $P_{ij}=P_{i}^{f}\cap P_{j}^{f}$ establishes the contact region between them. The true label $c_{p}^{*}$ of the fracture point is automatically generated based on the geometric distance across the fragments. For each point $p\in P_{i}$, $d_{p}$ indicates the minimum Euclidean distance from point *p* to any point *q* on the fragment $P_{j}$. If $d_{p}$ is less than the threshold τ, $c_{p}^{*}$ is marked as 1; otherwise, $c_{p}^{*}$ is marked as 0. This process is implemented by Formula (3):

(3) $$d_{p}=\sqrt{min(square\_distance(p,q)+{mask}_{diagonal})},$$

Here, $q\in P_{j}$, ${mask}_{diagonal}$ masks the distances between points within the same fragment.

The loss function employs weighted binary cross-entropy loss to address the issue of imbalanced positive and negative samples, specifically expressed as:

(4) $$L_{cla}=-\frac{1}{N}\sum p[w_{p}c_{p}^{*}logc_{p}+(1-c_{p}^{*})log(1-c_{p})],$$

Here, $w_{p}$ is the weight dynamically adjusted based on the ratio of positive and negative samples. $c_{p}$ is composed of two layers of lightweight MLPs, which map point features $f_{p}\in R^{D}$ to fracture probabilities, specifically expressed as:

(5) $$c_{p}=\sigma ({MLP}_{2}(ReLU({MLP}_{1}(f_{p})))),$$

Here, *MLP*_1_ and *MLP*_2_ are two-layer multilayer perceptrons; σ(·) is the Sigmoid activation function; and ReLU represents the rectified linear unit activation function. This design is not only computationally efficient but also effectively captures the local geometric characteristics of the fracture surface.

### 3.3. Multi-Fragment Assembly

In multi-fragment assembly tasks, this paper proposes a two-stage matching strategy that integrates global geometry-guided coarse matching with local geometry-constrained fine matching. Additionally, it incorporates differentiable optimization and dynamic weight learning mechanisms to facilitate the efficient joint matching across multiple fragments. The essence of the strategy lies in effectively reducing matching ambiguity: the coarse stage rapidly narrows down the search space by excluding fragment pairs with low global shape similarity, while the fine stage focuses computational resources on evaluating geometrically plausible candidates with high local feature affinity, thereby mitigating the risk of combinatorial explosion.

Regarding coarse matching: the global encoder generates global feature vectors by aggregating the spatial distribution information of keypoints. Based on this, the cosine similarity matrix $S_{coarse}\in R^{B\times B}$ for samples within a batch is calculated, specifically represented as:

(6) $$S_{coarse}[b_{1},b_{2}]=\frac{G[b_{1}]\cdot G[b_{2}]}{\left||G[b_{1}]||||G[b_{2}]|\right|},$$

Here, $G\in R^{B\times F}$ is the global feature vector with batch size B and dimension F; *b*_1_ and *b*_2_ are batch indices. This matrix reflects the overall geometric correlation between different fragments. To construct the coarse matching matrix $C\in R^{B\times N^{\prime }\times N^{\prime }}$, a masking mechanism is introduced to restrict the matching range. The masking generation rule is: if keypoints belong to the same fragment, the mask value is 0 (matching is prohibited); otherwise, it is 1 (matching is allowed). The coarse matching matrix is obtained by performing an element-wise multiplication of the Sigmoid normalization and the mask.

Regarding fine matching: The fine matching module extracts keypoint features $F_{b},F_{p}\in R^{B\times N^{\prime }\times d\;}$ from two directions using affinity network, and following this, calculates the local similarity matrix $S_{fine}\in R^{B\times N^{\prime }\times N^{\prime }}$ is specifically represented as:

(7) $$S_{fine}=AffinityLayer(F_{b},F_{p})⊙A,$$

Here, AffinityLayer is the affinity calculation layer, which computes the similarity score matrix by performing a dot product operation between features of multiple fragments (i.e., original dual descriptors) to enhance the expressive power of matching relationships between features; A is dynamically built for each pair of fragments based on their spatial proximity and the predicted fracture points. This guarantees that only points within a reasonable neighborhood can be matched. It enforces a fundamental physical constraint—matching points must be spatially close to form a valid connection—which is crucial for the overall physical consistency of the assembly.

Regarding staged fusion and differentiable optimization: Coarse and fine matching results are fused using dynamic weights α to generate a joint matching score matrix $S_{combined}$, specifically represented as:

(8) $$S_{combined}=\alpha C+(1-\alpha )S_{fine},$$

where the weight α is a learnable parameter that adaptively adjusts the contribution ratio between the two stages during training. To convert the score matrix into a probability distribution, the Sinkhorn algorithm is utilized to iteratively optimize the dual random matrix *X*∈${\left[0,1\right]}^{N\prime \times N\prime }$, with the update rule specifically expressed as:

(9) $$X^{\left(t+1\right)}={SoftMax}_{rows}\left(S_{combined}+\gamma {logX}^{\left(t\right)}\right),$$

(10) $$X^{\left(t+1\right)}={SoftMax}_{columns}\left(X^{\left(t+1\right)}\right)$$

Here, γ is the smoothing coefficient. After iteration until convergence, the soft matching matrix *X* is obtained, which is ultimately discretized into a binary matching matrix $X^{*}$ using the Hungarian algorithm; *X*(*t*) is the soft matching matrix of the t-th iteration.

The matching loss consists of four parts:

(1) Segmentation loss $L_{cls}$: This is calculated using a weighted cross-entropy function to supervise the keypoint classification results from the surface segmentation module.

(2) Fine matching loss $L_{fine}$: This component constrains the consistency between the soft matching matrix *X* and the true matching matrix $X^{gt}$:

(11) $$L_{fine}=-\sum_{i,j}X_{ij}^{gt}logX_{ij},$$

Here, $X_{ij}$ is the model-predicted soft matching matrix, which represents the matching probability between keypoints *i* and *j* (a double random matrix generated by the Sinkhorn algorithm using). $X_{ij}^{gt}$ is the true matching matrix (N′ × N′), where 1 indicates a match between points *i* and *j*, and 0 otherwise.

(3) Coarse Matching Loss $L_{coarse}$: This loss function constrains the coarse matching matrix C using mean squared error:

(12) $$L_{coarse}=||C-X^{gt}{\left|\right|}_{2}^{2},$$

where C is the coarse matching matrix of size B × N′ × N′, obtained by normalizing the global feature cosine similarity *S_coarse_* via the Sigmoid function (see Formula (6)).

(4) Rigidity loss $L_{rigid}$: This loss function is designed to ensure that matched point pairs adhere to consistency in rigid transformations. It is accomplished by minimizing the error in the rotation matrix and translation vector, thereby precisely constraining the rigid transformation relationship. The total loss is expressed as a weighted sum:

(13) $$L=\lambda_{cls}L_{cls}+\lambda_{fine}L_{fine}+\lambda_{coarse}L_{coarse}+\lambda_{rigid}L_{rigid},$$

Here, *L* is the total loss. $\lambda_{cls},{\;\lambda }_{fine},\;\lambda_{coarse},\;\lambda_{rigid}$ are the weighting coefficients of each loss term, which are used to balance the contributions of different tasks. $L_{cls},\;L_{fine},\;L_{coarse},\;L_{rigid}$ denote the four types of sub-losses.

### 3.4. Global Alignment

In 3D fragment assembly, global alignment estimation restores the pose of fragments within a unified coordinate system. This paper proposes an auxiliary transformation generation mechanism based on the geometric center of fracture point clouds. The mechanism consists of two parts: pairwise transformation and global transformation. The pairwise transformations utilize matching information from fracture points to calculate the rigid transformations between fragments, establishing an initial assembling relationship; However, for fragment pairs with insufficient matching information (such as isolated fragments), auxiliary edges are needed to enhance the integrity and accuracy of the assembly. Specifically, this mechanism constructs an initial transformation matrix (with the rotation component being the identity matrix) utilizing the translation vector derived from the geometric center of the point cloud within the fractured region to avoid biases caused by random initialization. This approach provides a physically meaningful starting point for optimization. Unlike random initialization, which can lead to divergent optimization trajectories, especially in symmetric structures or noisy scenes, the geometric center offers a stable and unbiased initial state. The initial state is already close proximity to the correct alignment, thereby significantly improving convergence stability and reducing cumulative errors. However, It is crucial to acknowledge that while centroid-based initialization provides a more stable and physically plausible starting point compared to random initialization, it remains vulnerable to local optima in cases of highly symmetric or repetitive fragment structures. Specifically, such configurations may allow multiple alignments to produce comparable geometric center overlaps. This represents a known challenge for global optimization techniques under these degenerate configurations.

Subsequently, the Shonan rotation averaging algorithm is employed to enhance the optimization of the global transformation, effectively suppressing cumulative errors. This mechanism significantly improves both the robustness and accuracy of the optimization process.

To support the aforementioned global alignment mechanism, pairwise transformation must first be performed. The goal of pairwise transformation estimation is to extract geometric alignment relationships from the matching matrix. Specifically, given fragments $P_{i}$ and $P_{j}$, the matching matrix $X_{ij}\in {\left\{0,1\right\}}^{N_{i}\times N_{j}}$ establishes the correspondence between keypoints of the two fragments. The set of matched point pairs $M=\{(p_{m},q_{n})|X_{mn}=1\}$ is obtained by extracting the indices of non-zero elements from the matrix $X_{ij}$. Subsequently, the RANSAC algorithm is used to robustly estimate the rigid transformation between fragments. RANSAC calculates candidate transformations by randomly sampling three sets of matched points, filters for inliers using a distance threshold (e.g., 0.05), and finally selects the transformation with the most inliers as the optimal estimation. It is important to note that this stage relies solely on local matching information and serves as the foundation for subsequent global optimization. When the number of matching point pairs is insufficient (e.g., |M|< 3), the corresponding edge calculation is skipped to avoid introducing low-quality constraints. In such cases, or when the global factor graph lacks sufficient connectivity, the previously proposed auxiliary transformation generation mechanism is applied to supplement the constraints.

Following the pair transformation, global transformation optimization is executed by integrating all pairwise constraints through the construction of a factor graph model and appropriately supplementing auxiliary edges to enhance graph connectivity. In factor graph, the vertices represent the global poses of each fragment $T_{i}$∈SE(3), and the edges contain two types of geometric constraints: (1) transformations estimated from fracture point matching; (2) auxiliary transformations based on geometric centers of fracture point clouds.

When the original edge set cannot guarantee the connectivity of the factor graph, auxiliary edges must be generated. The transformation matrix of auxiliary edges strictly follow the previously proposed method, calculated via geometric center alignment, specifically expressed as:

(14) $${\hat{T}}_{ij}^{aux}=\left[\begin{array}{cc} I_{3} & c_{j}-c_{i}\\ 0 & 1 \end{array}\right],$$

Here, $c_{i}$ and $c_{j}$ are the geometric centers of the keypoint in the fracture regions of the fragments $P_{i}$ and $P_{j}$; *I*_3_ is a 3 × 3 unit matrix.

As mentioned noted, the transformation shifts the geometric center of $P_{i}$ to align with the geometric center of $P_{j}$, thereby avoiding geometric contradictions resulting from randomly generated rotations and translations. In the optimization process, lower weights are assigned to auxiliary edges to weaken their influence, while ensuring the connectivity of the factor graph.

After constructing the factor graph that incorporates the aforementioned two types of constraints, global alignment is optimized through the Shonan average algorithm. The advantage of this algorithm lies in approximating the pose using its corresponding vector space representation, which simplifies the optimization problem by formulating it as a linear least squares problem that could be solved iteratively. The optimization aims to minimize the weighted residuals, specifically expressed as:

(15) $$\begin{array}{c} min\\ T_{1},\ldots ,T_{v} \end{array}\sum_{(i,j)\in E}{Log({\hat{T}}_{ij}^{-1}T_{i}^{-1}T_{j})}^{T}\Omega_{ij}Log({\hat{T}}_{ij}^{-1}T_{i}^{-1}T_{j}),$$

Here, *Ω_i_*_j_ is the weight matrix for transformation uncertainty (specifically for auxiliary edges $\Omega_{ij}^{aux}=10^{-4}I_{6}$); *E* contains the set of original and auxiliary edges. To eliminate coordinate system degrees of freedom, the pose of the largest fragment is designated as the reference frame, and its transformation matrix is fixed as $I_{4}$. Qwing to the aforementioned auxiliary transformation generation mechanism, experimental results demonstrate that the proposed method achieves a substantial reduction in global alignment error compared to randomly generating auxiliary transformations. In summary, this method addresses the matching constraints and graph connectivity issues by leveraging the geometric properties of fractured point clouds, thereby offering a high-precision solution for complex fractured scenarios, such as cultural relic restoration.

## 4. Experiment

### 4.1. Protocol

All experiments were conducted on a Linux workstation with the following hardware configuration: 2 NVIDIA GeForce RTX 3090 GPUs (each with 24 GB of VRAM), an Intel(R) Core(TM) i9-14900K CPU, and 125 GB of memory. The hardware and environmental configurations simulated in the experiments are shown in Table 1. The model parameter configurations include the optimizer learning rate (*η = 5$\;\times \;10^{-4}$*), dynamic weight initial value (α_0_ = 0.5), and Sinkhorn iteration count (T_iter_ = 50). These parameters were determined through a grid search on a validation set, with the objective of achieving an optimal balance among training stability, convergence speed, and final matching accuracy. The model was trained for 250 epochs until convergence, with an average training time of approximately 72 h on the specified hardware. The average inference time for assembling a pair of fragments is 0.3 s. For a complex scene involving a scene with 10 fragments, the entire end-to-end assembly process takes approximately 5 s. These performance metrics serve as compelling evidence of the practical efficiency exhibited by the GeoAssemble framework.

#### 4.1.1. Datasets

This paper evaluated our assembly model on both synthetic and real-world fragment datasets to comprehensively assess generalization capability under various complexities: (1) Breaking Bad [8], the largest synthetic fracture dataset for 3D assembly. It provides massive-scale data with diverse fracture patterns, enabling rigorous benchmarking of algorithmic robustness and scalability. The GeoAssemble network was trained on the everyday subset and tested on both the everyday and artifact subsets to ensure fair comparison with other methods. The everyday subset contains 498 objects and 41,754 fracture fragments. The training set consists of 34,075 fracture samples generated by 407 objects, and the test set includes 7679 fracture samples from 91 objects. The artifact subset consists of 3651 fracture samples generated by 40 unclassified objects. (2) Fantastic Breaks [37], a real-world dataset of 195 manually scanned fractured objects with complex surfaces, used for testing only. Its fragments exhibit real-world challenges like noise, erosion, and material variations, testing the practical applicability of the method beyond synthetic benchmarks.

#### 4.1.2. Evaluation Metrics

This study adopted three evaluation criteria to assess assembly quality. These criteria encompass the mean absolute error (MAE) and root mean square error (RMSE) of rotation and translation in global alignment, as well as the part alignment accuracy (PA) metric. rotation (MAE/RMSE(R)) and translation (MAE/RMSE(T)) errors directly quantify the precision of pose estimation in degrees and meters, respectively. PA measures whether the average chamfer distance between each point in the assembly result and the ground-truth model is less than 0.01, thereby reflecting the proportion of fragments that have been accurately and completely assembled.

#### 4.1.3. Baseline Methods

In this study, we adopt the recently proposed Jigsaw [38] framework as main benchmark. This method is specifically designed for multi-fragment assembly tasks, significantly improving assembling accuracy by jointly learning of fragment segmentation and point matching. For comprehensive comparison, we include several state-of-the-art approaches: DGL utilized an iterative graph neural network to infer relationships among fragments; PHFormer [39] proposed an agent-level hybrid Transformer with hierarchical attention for fragment relationships modeling; GPAT [10] designs a geometric point attention mechanism that explicitly encodes local rigid transformations into feature learning. All baseline methods use textureless point clouds as input and are trained on the everyday subset of the Breaking Bad dataset.

### 4.2. Multi-Fragment Assembly

#### 4.2.1. Breaking Bad

Table 2 present the quantitative comparison results of different methods on the different datasets. The results demonstrate that GeoAssemble significantly outperforms previous SOTA methods. On the Everyday dataset, GeoAssemble attains an average rotation error of 34.0°, marking a 17% improvement relative to Jigsaw, while achieving a translation error of 6.31 × 10^−2^, an 18% reduction compared to the best-performing baseline. Additionally, GeoAssemble successfully restored 71% of fragments to their original poses, while Jigsaw achieved only 63%. On the Artifact subset, GeoAssemble maintains a robust part alignment accuracy (PA) of 46%, clearly showcasing its superior cross-category generalization capabilities and adaptability to diverse object geometries.

**Figure 3:**
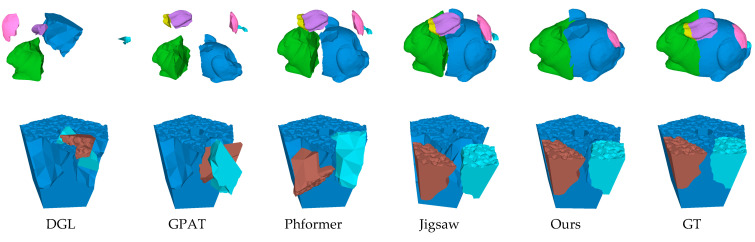
The visualization results of different methods on the Artifact dataset.

**Figure 4:**
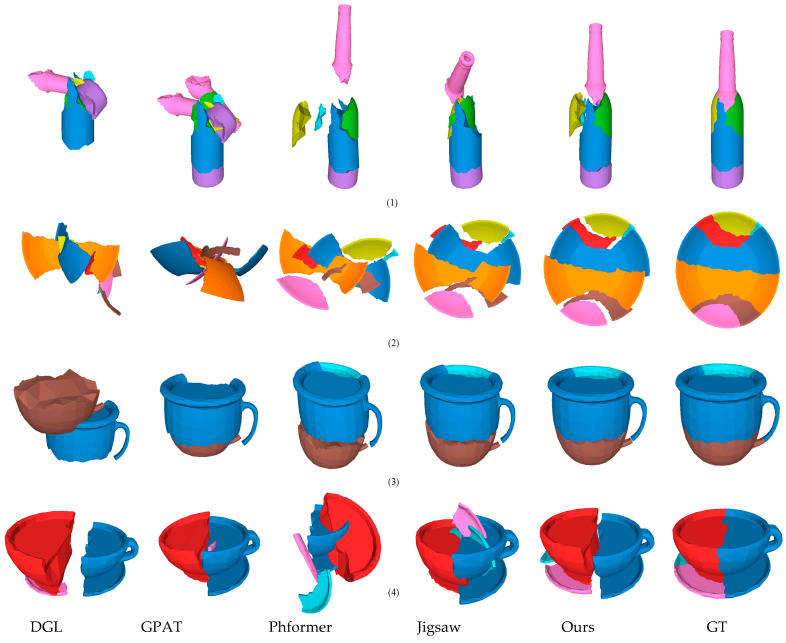
The visualization results of different methods on the Everyday dataset.

As shown in Figure 3 and Figure 4, the visualization results indicate that GeoAssemble achieves significantly higher pose estimation accuracy than previous SOTA methods. In addition, as shown in Figure 4(2), GeoAssemble still maintain high assembly accuracy even when confronted with typically challenging scenarios such as thin-walled objects. This superiority is evident in the seamless alignment of fracture surfaces, a task which other methods consistently struggle: DGL’s iterative graph update mechanism frequently converges to suboptimal solutions; GPAT’s local geometric attention lacks sufficient global context, leading to misalignments in complex structures; Despite the robust performance of PhFormer remains susceptible to interference from ambiguous feature matches; and Jigsaw’s reliance on pre-segmented parts limits its flexibility in geometry-only assembly scenarios.

The performance improvement stems from three key innovations: its geometry-enhanced feature encoding, which delivers robust discriminative capabilities to minimize mismatches; a two-stage matching mechanism that effectively reduces the combinatorial search space; and geometry-centered initialization that ensures stable convergence toward a global optimal solution. Notably, GeoAssemble operates in a category-agnostic manner, relying exclusively on geometric features without requiring object labels or category-specific knowledge.

#### 4.2.2. Fantastic Breaks

Table 3 presents the quantitative comparison results of different methods on the Fantastic Breaks dataset. The results indicate that GeoAssemble consistently outperforms baseline methods. GeoAssemble achieves an average rotation error of 22.1°, representing a 41% reduction compared to Jigsaw. It also delivers a translation error of 6.2 × 10^−2^, which is 36% lower than the best baseline result. Additionally, GeoAssemble successfully restores 71% of fragments to their original poses, while Jigsaw achieves only 54%.

**Figure 5:**
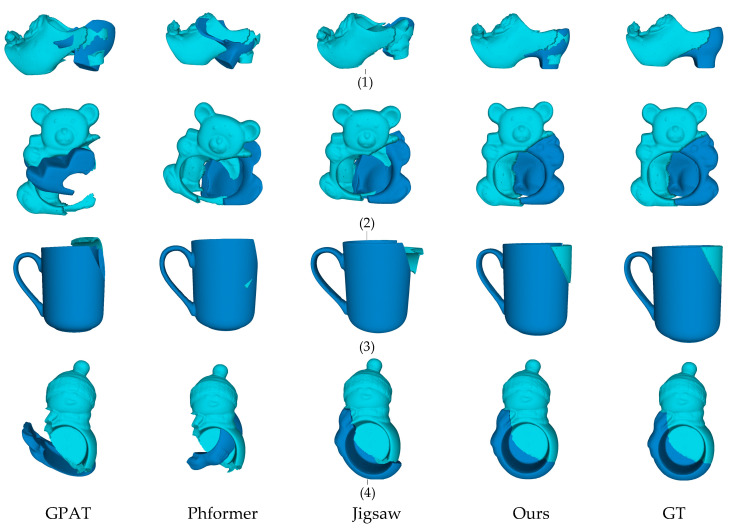
The visualization results of different methods on the Fantastic Breaks dataset. In this figure, (1)–(4) represent the assembly results of fragments with different materials.

Figure 5 show comparative assembly results on a real-world fracture dataset, validating the practical effectiveness of the GeoAssemble. The visualization results clearly illustrate that our model can successfully handle the highly irregular geometric shapes of the fragments, the complex erosion patterns on fracture surfaces, and the cross-material generalization requirements. For example, when assembling broken ceramic fragments (see Figure 5(3)), GeoAssemble exhibits superior matching accuracy compared to baseline methods, successfully overcoming the challenge of capturing real-world fracture geometric features that often elusive in synthetic datasets (see Breaking Bad) due to simulation limitations or regularized fractures. In contrast, the baseline methods struggle with these real-world imperfections: the noise and erosion often disrupt the local feature matching schemes of GPAT and PhFormer, while the highly irregular shapes challenge the global assumptions of DGL and Jigsaw.

#### 4.2.3. Ablation Experiments

To evaluate the performance of the core modules within the GeoAssemble framework, this paper presents a series of hierarchical ablation experiments conducted on the Breaking Bad dataset. In these experiments, PointNet++ serves as the baseline feature extractor, which is combined with a single-stage matching and a randomly initialized auxiliary edge configuration. The detailed results are presented in Table 4.

**Figure 6:**
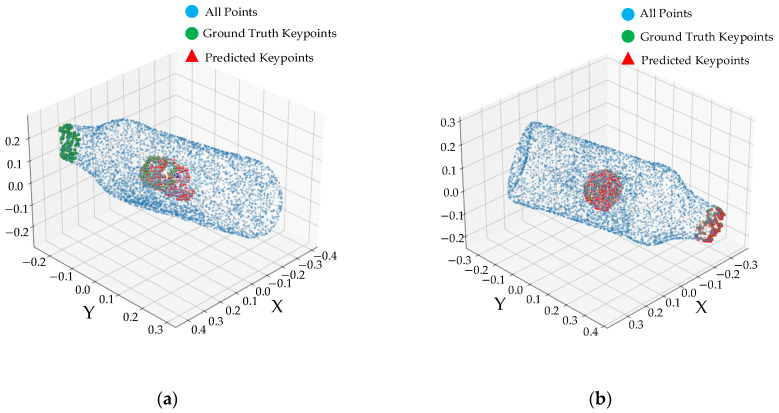
Fracture point recognition of Jigsaw and GeoAssemble. Specifically, Subfigure (**a**) and Subfigure (**b**) respectively display the fracture point recogniton results from the Jigsaw model and the GeoAssemble model.

As shown in Table 4, the baseline model achieves only 63.6% PA on the Everyday subset, with a MAE-R as high as 35.6°. This result exposes the significant limitations of the baseline model: the static sampling strategy struggles to adequately capture geometric variations on fracture surfaces, single-stage matching suffers from combination explosion due to the lack of global constraints, and randomly initialized auxiliary edges accumulate errors in symmetric structures. Conversely, the enhanced DGCNN network in this paper, which integrates centroid relative position features, significantly reduces the rotation error by 16% (with MAE-R decreases to $29.9^{{}^{\circ}}$). Figure 6 shows the corresponding fracture point prediction results, where Figure 6a,b present those of the baseline model and GeoAssemble model, respectively. In these figures, blue, green, and red point clouds represent ordinary points, actual fracture points, and predicted fracture points, respectively. Notably, it is evident that some actual fracture points have not been predicted in Figure 6a, whereas, nearly all of them have been successfully predicted in Figure 6b. The above clearly demonstrates the effectiveness of dynamic neighborhood construction and spatial context enhancement in extracting geometric details.

As shown in Table 4, the baseline model achieves only 63.6% PA on the Everyday subset, with a MAE-R as high as $35.6^{{}^{\circ}}$. This result exposes the significant limitations of the baseline model: the static sampling strategy struggles to adequately capture geometric variations on fracture surfaces, single-stage matching suffers from combination explosion due to the lack of global constraints, and randomly initialized auxiliary edges accumulate errors in symmetric structures. Conversely, the enhanced DGCNN network in this paper, which integrates centroid relative position features, significantly reduces the rotation error by 16% (with MAE-R decreases to $29.9^{{}^{\circ}}$). Figure 6 shows the corresponding fracture point prediction results, where Figure 6a,b present those of the Jigsaw model and GeoAssemble model, respectively. In these figures, blue, green, and red point clouds represent ordinary points, actual fracture points, and predicted fracture points, respectively. Notably, it is evident that some actual fracture points have not been predicted in Figure 6a, whereas, nearly all of them have been successfully predicted in Figure 6b. The above clearly demonstrates the effectiveness of dynamic neighborhood construction and spatial context enhancement in extracting geometric details.

The two-stage matching strategy further improves the performance of our model: coarse matching filters candidate pairs by leveraging cosine similarity based on keypoint aggregation, while fine matching optimizes candidate pairs by combining local feature similarity with the Sinkhorn algorithm. This dual method achieves a 67% successful alignment rate for Chamfer distances < 0.01, representing a 4.3% improvement over the baseline. Geometric center initialization replaces random transformations by calculating the centroid *c_i_* of fracture regions to generate the initial translation vector *t_ij_ = c_j_ − c_i_*, while employing the Kabsch algorithm to solve for the rotation matrix. This initialization achieves a 71% successful alignment rate for symmetric objects. The complete model delivers a segmentation accuracy of 46.6% on the artifact subset (a 10% improvement over the baseline), demonstrating the synergistic effects of dynamic features, hierarchical matching, and geometric initialization. These innovations collectively provide an efficient closed-loop solution for complex fracture scenarios.

## 5. Conclusions

This paper proposes a geometry-aware 3D fragment assembly framework that combines multi-dimensional feature representation with physical constraint optimization, achieving substantial improvements in assembly accuracy and robustness. Specifically, the framework employs a DGCNN network that integrates local geometric features to enhance detail representation capability in fractured regions; a keypoint-guided staged matching strategy balancing global and local optimization; and a geometric center initialization method reducing pose estimation errors in global alignment. Experimental results demonstrate that our model performs exceptionally well in complex fractures, symmetrical structures, and noisy scenarios, without relying on semantic priors, validating its applicability for cultural heritage restoration and industrial quality inspection. The annotation-free nature of our method makes it particularly suitable for archaeological fragment reassembly, where semantic labels are unavailable. Furthermore, while the current framework demonstrates efficient performance on scenes with up to 20 fragments, its scalability to very large-scale assemblies (e.g., >50 fragments) requires further investigation, particularly in optimizing the global alignment step.

However, it exhibits insufficient geometric sensitivity for thin-shell fractured objects, which may lead to minor edge misalignments. Future research will focus on optimizing the robustness of thin-shell object reconstruction, exploring cross-modal data fusion and self-supervised learning. Furthermore, its robustness to noise and ability to handle complex geometries suggest potential applications in medical imaging, such as virtual reconstruction of fractured bones or anatomical structures from segmented scans.

## Figures and Tables

**Table 1:** Experimental environment.

Configuration Name	Parameter
CPU	Intel(R) Core(TM)i9-14900K
GPU	NVIDIA GeForce RTX 3090
CUDA	11.3
Pytorch	1.10.1 + cu113

**Table 2:** The quantitative results of baseline methods and GeoAssemble on the Breaking Bad dataset.

Method	RMSE(R) Degree ↓	MAE(R) Degree ↓	RMSE(T) $\times \;10^{-2}\;↓$	MAE(T) $\times \;10^{-2}\;↓$	PA% ↑
Tested on the Everyday dataset					
DGL [40]	82.3	68.7	16.2	13.6	23.7
GPAT [10]	79.3	66.4	14.4	11.1	30.2
Phformer [39]	34.4	29.4	10.0	8.1	47.4
Jigsaw [38]	41.2	35.6	7.7	6.12	63.6
GeoAssemble	34.0	29.4	6.3	4.98	71.1
Tested on the Artifact dataset					
DGL [40]	85.4	76.2	18.7	15.4	7.2
GPAT [10]	78.7	73.4	16.2	13.6	9.3
Phformer [39]	36.5	31.8	13.2	11.4	21.9
Jigsaw [38]	57.9	50.2	16.9	13.8	36.5
GeoAssemble	50.3	43.4	14.1	11.5	46.6

**Table 3:** The quantitative results of baseline methods and GeoAssemble on the Fantastic Breaks dataset.

Method	RMSE (R) Degree ↓	MAE (R) Degree ↓	RMSE(T) $\times \;10^{-2}\;↓$	MAE(T) $\times \;10^{-2}\;↓$	PA% ↑
GPAT [10]	72.7	62.3	15.6	13.4	37.2
Phformer [39]	39.3	32.2	14.4	11.7	45.7
Jigsaw [38]	43.1	37.8	9.7	7.8	54.6
GeoAssemble	26.0	22.1	6.2	4.9	71.3

**Table 4:** The ablation study result of GeoAssemble.

Components				RMSE (R)	MAE (R)	RMSE (T)	MAE (T)	PA
DGCNN	Centroid Position	Matching	Auxiliary Edges	Degree ↓	Degree ↓	$\times 10^{-2}\;↓$	$\times 10^{-2}\;↓$	% ↑
				41.2	35.6	7.77	6.12	63.6
**√**				35.6	31.2	6.98	5.54	65.2
**√**	**√**			34.8	29.9	6.77	5.31	66.7
**√**	**√**	**√**		34.5	29.8	6.69	5.29	67.9
**√**	**√**	**√**	**√**	34.0	29.4	6.31	4.98	71.1

## Data Availability

The data that support the findings of this study are publicly available. The synthetic fragment dataset (Breaking Bad) can be accessed on 20 October 2022 at [https://breaking-bad-dataset.github.io/]. The real-world fragment dataset (Fantastic Breaks) can be accessed on 22 June 2023 at [https://terascale-all-sensing-research-studio.github.io/FantasticBreaks/].
